# Neutrophil-to-lymphocyte ratio is an independent predictor for neurological disability in patients with idiopathic transverse myelitis

**DOI:** 10.1186/s12883-023-03384-3

**Published:** 2023-09-25

**Authors:** Je Hong Min, Sung-Yeon Sohn, Seung Yeon Lee, Sang Hyun Seo, Shin Yeop Kim, Bumhee Park, Seung Il Kim, In Soo Joo

**Affiliations:** 1 Department of Neurology, Ajou University School of Medicine, Ajou University Medical Center, 164, World Cup-Ro, Yeongtong-Gu, Suwon, Republic of Korea; 2 Ajou University School of Medicine, Suwon, Republic of Korea; 3 Department of Biomedical Informatics, Ajou University School of Medicine, Suwon, Republic of Korea; 4 Office of Biostatistics, Medical Research Collaborating Center, Ajou Research Institute for Innovative Medicine, Ajou University Medical Center, Suwon, Republic of Korea

**Keywords:** Transverse myelitis, Neutrophil-to-lymphocyte ratio, Neurological disability, Prognostic factor

## Abstract

**Introduction:**

The neutrophil-to-lymphocyte ratio (NLR) has been found to be useful in the prognostication of immune-mediated neurological disorders because it roughly reflects the systemic innate immune response compared to the adaptive immune response. However, studies on the validity of NLR in demyelinating disorders of the central nervous system have shown conflicting results. Therefore, we aimed to investigate NLR in the idiopathic transverse myelitis (ITM) cohort.

**Methods:**

We retrospectively analyzed the cohort data of patients with ITM between January 2006 and February 2020. The medical data of all patients with myelitis were reviewed to exclude patients with disease-associated myelopathy according to predefined exclusion criteria. The relationship between the natural log-transformed NLR (lnNLR) and the clinical, paraclinical, and imaging data was evaluated. Factors associated with neurological disability were analyzed using a linear mixed-effects model. Predictive factors for moderate-to-severe neurological disability (Expanded Disability Status Scale [EDSS] score ≥ 4) were investigated.

**Results:**

A total of 124 participants were included in the analysis. The lnNLR correlated with EDSS and lesion length. Linear mixed-effects analysis showed that age, lesion length, and lnNLR were independently associated with neurological disabilities. Multivariable logistic regression revealed that lnNLR (odds ratio [OR] = 4.266, 95% confidence interval [CI] = 1.220–14.912, *p* = 0.023) and lesion length (OR = 1.848, 95% CI = 1.249–2.734, p = 0.002) were independent predictive factors of the worst neurological disability.

**Conclusion:**

NLR may be used as an independent prognostic factor for predicting poor neurological outcomes in patients with ITM.

## Introduction

Transverse myelitis (TM) is a neuroimmunological disorder caused by spinal cord inflammation. The specific etiology of TM has not been identified in many patients despite thorough diagnostic evaluations. These patients are referred to as having idiopathic TM (ITM) [1]. ITM is thought to represent a distinct feature even in the “post-aquaporin-4 (AQP4) antibody era” [2], especially in East Asia, where the incidence of multiple sclerosis and neurosarcoidosis is very low.

Evidence from early research has supported the role of systemic inflammation in ITM [3, 4]. A history of antecedent infection [5] or post-vaccination [6] in ITM may also support this notion. The neutrophil-to-lymphocyte ratio (NLR) is an easily obtained index derived from routine blood tests and is considered useful as a marker of the innate immune response in the body [7]. The NLR has been studied as a useful predictive factor of clinical outcomes in central nervous system (CNS) demyelinating disorders [8–11] as well as in cancer [12], and stroke [13]. However, studies on the validity of NLR in various CNS demyelinating disorders, including multiple sclerosis (MS) [7–9], neuromyelitis optica spectrum disorders (NMOSD) [10, 11, 14], and myelin oligodendrocyte glycoprotein antibody-associated disease (MOGAD) [15], have shown conflicting results.

We investigated the prognostic role of NLR in our large ITM cohort. The NLR has not been evaluated earlier in patients with ITM. We also explored the relationship between NLR and other clinical and paraclinical factors using statistical analyses of various facets.

## Patients and Method

### Study design and ethical approval

This retrospective study was conducted in a tertiary university-affiliated hospital. This study was performed in accordance with the Declaration of Helsinki and was approved by the by the Institutional Review Board of Ajou University Hospital (MED-MDB-20–033). The requirement for informed consent was waived by ethics committee of Ajou University Hospital, because of the retrospective nature of the study.

### Patients

To identify all patients with ITM, the participants were selected according to the following process. The medical records and magnetic resonance imaging (MRI) of all patients between January 2006 and February 2020 with diagnostic codes including “myelopathy,” “myelitis,” “acute transverse myelitis in demyelinating diseases of CNS,” “encephalitis, myelitis or encephalomyelitis,” “neuromyelitis optica,” multiple sclerosis” were initially reviewed by the authors (S.Y.K. and S.-Y.S.). Patients with isolated myelitis of suspected inflammatory etiology were selected for this study. Among the selected inflammatory myelitis patients, the follow-up medical records were reviewed to include clinically (1) development of symptoms attributable to the spinal cord; (2) defined sensory level and bilateral symptoms; (3) symptoms onset to reach maximal progression between 4 h and 21 days, with (4) cerebrospinal fluid (CSF) pleocytosis or elevated immunoglobulin G (IgG) index or gadolinium enhancement, and to exclude (1) disease-associated TM secondary to MS, NMOSD, acute disseminated encephalomyelitis, rheumatoid/connective tissue disorders (e.g., systemic lupus erythematosus, neurosarcoidosis), infectious/parainfectious diseases (e.g., herpes zoster, human T-cell leukemia virus-1 [HTLV-1]), metabolic disorders (e.g., cobalamin deficiency); (2) MRI evidence of potential demyelinating brain lesions at admission or during follow-up; (3) conditions that may affect NLR (e.g., concomitant untreated systemic infection, malignancy in the last 30 days, or previously confirmed autoimmune comorbidities). Patients (4) aged < 18 years, (5) with a short follow-up period (< 3 months), (6) with a history of steroid administration within 2 weeks before blood sampling, or (7) with no available white blood cell differential count were further excluded.

### Clinical assessment

The baseline characteristics of the patients, date of symptom onset, follow-up duration, and presumed etiology were documented. Patients were defined as having acute TM if they reached a clinical nadir within 3 weeks of symptom onset [1]. Neurological disability was assessed using a modified version [16] of Kurtzke Expanded Disability Status Scale (EDSS) [17]. The EDSS at initial admission, clinical nadir, and at 3-month, 1- and 2-year follow-ups were assessed. The worst EDSS was the EDSS at nadir in most monophasic patients, except for relapsing cases. Neurological outcomes were dichotomized as moderate to severe disability (EDSS ≥ 4) or severe disability (EDSS ≥ 6), since these have been previously considered important milestones [10, 11].

### Laboratory tests

#### Serological evaluation

Complete blood counts with differential counts were analyzed from samples immediately drawn at the time of admission. Extensive serological evaluation was performed, including vitamin B12, folate, copper, venereal disease, HTLV-1, human immunodeficiency virus, rheumatoid factor, antinuclear antibody, antineutrophil cytoplasmic antibody, angiotensin-converting enzyme, paraneoplastic antibodies, parasitic antibodies including *Toxocara canis* IgG, total IgE, *Dermatophagoides pteronyssimus*, and *Dermatophagoides farinae*, and evaluation for viral infection or other parainfectious etiologies if clinically indicated. The timing of blood collection from the initial symptom onset (onset-to-sample time) within 3 weeks was arbitrarily defined as “acute-phase” samples.

#### Autoantibody testing and CSF study

IgG antibody testing against AQP4 was performed using indirect immunofluorescence on a substrate of mouse cerebellum and midbrain (before 2017) as described earlier [18], and using a commercial cell-based assay (EUROIMMUN, Lübeck Germany) (since 2017). In patients with relapse, serial assays were performed on each admission before immunosuppressive therapy. The CSF study results were collected from all available subjects. The CSF data included cell count with differential, protein, glucose, virology (if indicated), IgG index, and oligoclonal bands.

#### Neuroimaging evaluation

Brain and whole spine MRIs (GE SIGNA™ HDxt 1.5 T scanner or GE Discovery™ MR750w 3.0 T scanner) were conducted in all patients. Those patients with suspected demyelinating brain lesions were excluded from this study. MRI lesion length was independently measured as vertebral body segments of the longest hyperintense lesion on sagittal T2-weighted images by two authors (S.Y.K. and S.-Y.S.). Any discrepancies were resolved through discussion to reach a consensus.

## Statistical analysis

Continuous variables are expressed as mean ± standard deviation (SD) or median (interquartile range [IQR] Q1–Q3), as appropriate. The frequency of categorical variables are expressed as a percentage (%). Normality was assessed using the KolmogorovSmirnov test. The distribution of NLR values was skewed to the right; therefore, we used the natural log-transformed NLR (lnNLR) [8]. After log transformation, lnNLR met the normality criteria. The Pearson’s or Spearman’s correlation statistics were used to assess the correlation between continuous variables or ordinal scales (e.g., EDSS). Linear associations between various variables and the lnNLR were explored using multivariable linear regression analysis. Neurological disability outcomes measured by repeated EDSS assessments were analyzed using a linear mixed-effects regression model with a patient-specific random intercept and slope for each patient. After exploring each potential predictive factor separately in the univariable analysis, multivariable models were constructed, including covariates such as demographic factors, onset-to-sample time, recurrence (for adjustment), and other significant variables in the univariable analysis. Multivariable binary logistic regression models were constructed to delineate independent predictive factors for the worst dichotomous neurological disability.

All statistical analyses were performed using SPSS version 25.0 (IBM Corporation, Armonk, USA) or R version 4.1.0 (http://www.R-project.org). Statistical significance was set at *P* < 0.05.

## Results

**Fig. 1:**
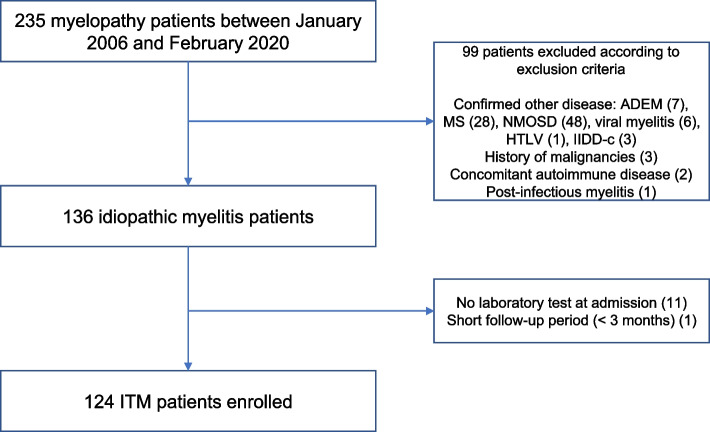
Flowchart showing ITM patients inclusion. *ITM* idiopathic transverse myelitis, *ADEM* acute disseminated encephalomyelitis, *MS* multiple sclerosis, *NMO* (seropositive) neuromyelitis optica, *NMOSD* (seronegative) neuromyelitis optica spectrum disorder, *HTLV* Human T-lymphotrophic virus type 1, *IIDD-c* idiopathic inflammatory demyelinating disease of the central nervous system

Among the initially identified 235 patients with myelopathy, 99 with disease-associated myelopathy or other comorbidities were excluded. After further excluding a small number of patients without laboratory data at admission or without an adequate follow-up period, 124 subjects were finally included in the idiopathic myelitis group (Fig. 1).

Demographic, clinical, and paraclinical characteristics are summarized in Table 1. One-twenty-four study subjects (85 men, 68.5%) were analyzed for a median follow-up period of 4.0 (IQR 1.0–8.7) years. Mean age was 45.4 ± 12.8 years with a median EDSS of 2.25 (IQR 2.0–3.0). Fifteen patients (12%) relapsed. The median MRI lesion length was 2.0 (IQR 1.5–3.5) vertebral segments; 50 (40.3%) patients had longitudinally extensive TM (LETM, defined as lesion length of three or more vertebral segments); myelitis lesions were most commonly located in the thoracic cord level (60.5%). Median white blood cell count was 7300/μL (IQR 6125.0–9277.5/μL); median NLR was 1.717 (IQR 1.304–2.452). CSF-specific oligoclonal bands were not identified in most subjects (*n* = 120, 96.8%), and serum anti-AQP4 antibody was negative in all subjects.

**Table 1:** Baseline characteristics and demographic data of the study subjects

Parameters	ITM group
N	124
Age (years)	45.4 ± 12.8
Sex	
Male	85 (68.5%)
Female	39 (31.5%)
Follow-up period (years)	4.0 (1.0–8.7)
EDSS	2.0 (1.5–3.5)
EDSS ≥ 4	20 (16.1%)
EDSS < 4	104 (83.9%)
MRI lesion location	
Cervical cord only	33 (26.6%)
Cervicothoracic cord	11 (8.9%)
Thoracic cord only	75 (60.5%)
Thoracolumbar cord	5 (4.0%)
MRI lesion length (vertebral segments)	2.0 (1.5–3.5)
WBC count (total, /µL)	7300.0 (6125.0–9277.5)
Neutrophil (%)	55.5 (48.0–63.2)
Lymphocyte (%)	32.9 (26.2–36.6)
NLR	1.717 (1.304–2.452)
lnNLR	0.625 ± 0.523
Immunoglobulin G index	0.524 (0.450–0.641)
Oligoclonal band	4 (3.2%)

*ITM* idiopathic transverse myelitis. *EDSS* expanded disability status scale, *MRI* magnetic resonance imaging, *WBC* white blood cell, *NLR* neutrophil-to-lymphocyte ratio, *lnNLR* natural log-transformed neutrophil-to-lymphocyte ratio

Patients who visited the hospital during the acute phase showed significantly higher initial EDSS (median 3.0 [IQR 2.0–3.75] vs. 2.0 [IQR 2.0–2.5]; Mann–Whitney test, *p* < 0.001), the worst EDSS (median 3.0 [IQR 2.0–4.25] vs. 2.0 [IQR 2.0–3.0]; Mann–Whitney test, *p* < 0.001), and NLR (2.880 ± 2.079 vs. 1.890 ± 1.104; t-test for lnNLR, *p* < 0.001). Age (*p* = 0.470) and lesion length (*p* = 0.858) showed no significant differences between the groups.

### Correlation analysis and linear associations between lnNLR and other variables

**Fig. 2:**
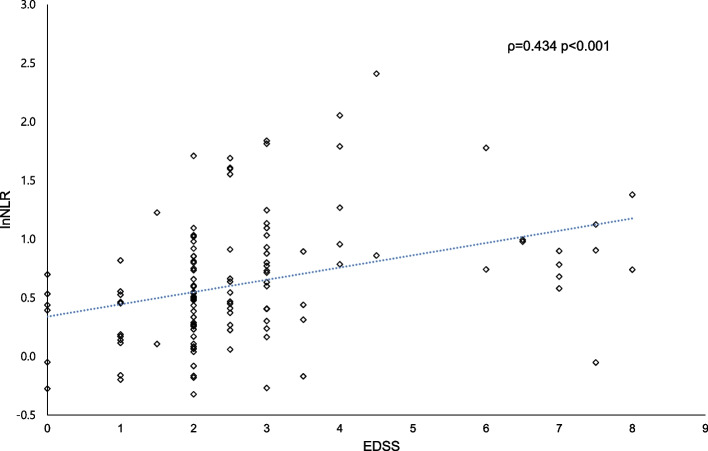
Correlation of the lnNLR with EDSS. *lnNLR* natural log-transformed neutrophil-to-lymphocyte ratio, *EDSS* expanded disability status scale

Correlation analysis revealed a positive correlation between neurological disability and lnNLR (Spearman’s ρ = 0.434, *p* < 0.001) (Fig. 2). A weak but significant correlation was observed between lnNLR and lesion length (Pearson’s *r* = 0.224, *p* = 0.012). Univariable regression analysis (lnNLR as the dependent variable) revealed a significant association with the worst EDSS, onset-to-sample time, and lesion length. Multivariable regression analyses were performed with adjustments for possible confounders (age, sex, and onset-to-sample time). Multivariable regression analysis revealed a significant association between the worst EDSS (*p* = 0.003) and onset-to-sample time (model 1), but the worst EDSS failed to reach statistical significance (*p* = 0.053) in a different model (model 2) (Table 2).

**Table 2:** Linear association with lnNLR as the dependent variable using linear regression analysis

	Univariable		Multivariable		Multivariable	
			model 1		model 2	
	beta	*p*	beta	*p*	beta	*p*
Age	0.037	0.680	-0.011	0.897	-0.001	0.993
Sex (F)	0.044	0.629	-0.058	0.519	-0.068	0.456
Onset-to-sample time	0.310	< 0.001^***^	0.242	0.012^*^	0.265	0.007^**^
Lesion length	0.224	0.012^*^	–	–	0.122	0.216
Worst EDSS	0.348	< 0.001^***^	0.273	0.003^**^	0.205	0.053

Variables included in multivariable model 1 (age, sex, worst EDSS, onset-to-sample time) and multivariable model 2 (age, sex, MRI lesion length, worst EDSS, onset-to-sample time) were selected from significant variables in the univariable analysis as well as adjusted variables

*lnNLR* natural log-transformed neutrophil-to-lymphocyte ratio, *F* female sex (compared to male), *EDSS* expanded disability status scale

^*^ *p* < 0.05; ^**^p < 0.01, ^***^p < 0.001

### Factors associated with neurological disability

Univariable analysis using a linear mixed-effects model with a patient-specific random intercept and slope for each patient revealed that age, onset-to-sample time, MRI lesion length, and lnNLR (*p* = 0.001) were significantly associated with repeatedly measured EDSS. Multivariable analysis showed that the lnNLR was independently associated with neurological disability when adjusting for age, sex, onset-to-sample time, recurrence, and MRI lesion length as covariates (*p* = 0.037) (Table 3). Further exploration with inclusion of the interaction term (time × lnNLR) in the linear mixed-effects model did not show significance, suggesting that lnNLR was not associated with time.

**Table 3:** Factors associated with neurological disability assessed by EDSS

	Univariable		Multivariable		
	Estimate	*p*	Estimate	95% CI	*p*
Age	0.024	0.017^*^	0.023	0.006–0.040	0.008^**^
Sex (F)	0.074	0.794	-0.365	-0.861–0.132	0.148
Onset-to-sample time	1.023	< 0.001^***^	0.895	0.369–1.422	0.001^**^
Recurrent disease	0.307	0.433	0.233	-0.432–0.898	0.489
Lesion length	0.335	< 0.001^***^	0.297	0.176–0.418	< 0.001^***^
lnNLR	0.957	< 0.001^***^	0.484	0.031–0.937	0.037^*^

Repeated EDSS assessments at each visit were set as the outcome, and were analyzed using linear mixed-effects regression model with subject-specific random intercept and slope for each subject. Variables included in the multivariable model (age, sex, onset-to-sample time, recurrent disease, MRI lesion length, lnNLR) were selected from significant variables in the univariable analysis as well as adjusted variables

*EDSS* expanded disability status scale, *CI confidence interval, F* female sex (compared to male), *lnNLR* natural log-transformed neutrophil-to-lymphocyte ratio

^*^p < 0.05; ^**^p < 0.01, ^***^p < 0.001

### Predictive factors for poor neurological outcome

We aimed to investigate NLR as an independent predictive factor for the worst neurological disability. Logistic regression analysis was performed to assess the independent predictive factors for predefined dichotomous neurological disability. Significant predictors for the worst outcome from univariable analyses of each variable were onset-to-sample time, MRI lesion length, and lnNLR. Multivariable analysis (including age, sex, and onset-to-sample time for adjustment) revealed that lnNLR (odds ratio [OR] = 4.266, 95% confidence interval [CI] = 1.220–14.912, *p* = 0.023) and lesion length (OR = 1.848, 95% CI = 1.249–2.734, *p* = 0.002) were independent predictive factors for moderate-to-severe neurological disability (EDSS ≥ 4) (Table 4). However, only the MRI lesion length (OR = 1.805, 95% CI = 1.273–2.559, *p* = 0.001) was found to be significant when severe neurological disability (defined as EDSS ≥ 6) was set as the outcome. Other variables, including recurrent disease and CSF profiles, showed no statistical significance; therefore, they were excluded from the final model.

**Table 4:** Predictive factors for worse neurological disability of EDSS 4 or more

	Univariable		Multivariable		
	Exp(B)	*p*	Exp(B)	95% CI	*p*
Age	1.035	0.078	1.043	0.993–1.097	0.095
Sex (F)	2.586	0.056	1.079	0.283–4.111	0.915
Onset-to-sample time	8.217	< 0.001^***^	9.141	2.113–39.549	0.003^**^
Recurrent disease	0.714	0.674	–	–	–
Lesion length	1.642	0.001^**^	1.848	1.249–2.734	0.002^**^
lnNLR	6.805	< 0.001^***^	4.266	1.220–14.912	0.023^*^

Worst EDSS was dichotomized as moderate to severe disability (EDSS ≥ 4) or mild (EDSS less than 4). Worst EDSS was the EDSS assessed at nadir (in monophasic patients), or at recur or at last follow-up (in recurrent patients). Variables included in the multivariable model (age, sex, onset-to-sample time, MRI lesion length, lnNLR) were selected from significant variables in the univariable analysis as well as adjusted variables. Nagelkerke pseudo R-squared value of the final multivariable model was 0.498

*EDSS* expanded disability status scale, *CI* confidence interval,* F* female sex (compared to male), *lnNLR* natural log-transformed neutrophil-to-lymphocyte ratio

^*^p < 0.05; ^**^p < 0.01, ^***^p < 0.001

## Discussion

Our results provide evidence for the clinical usefulness of NLR in patients with ITM. To the best of our knowledge, NLR has not been evaluated in patients with ITM. The ITM was diagnosed according to the criteria defined by the Transverse Myelitis Consortium Working Group [1]. Because ITM is a diagnosis of exclusion, it is not uncommon to reclassify it as a disease-associated form in many cases [19, 20]. We tried to recruit participants with the application of stringent criteria for ITM, that is, to include ‘truly idiopathic’ TM as much as possible. The retrospective nature of the study enabled us to follow-up the patients for a long period of time (median, 4 years), and therefore to exclude patients with disease-associated relapsing TM. Despite lack of disease-specific diagnostic measures, ITM denotes a distinct group of immune-mediated disease with a male predominance and a high proportion of monophasic course [2, 21], similar to the clinical characteristics of our cohort.

We found that NLR, as well as MRI lesion length, could serve as an independent predictive factor for unfavorable neurological disability. This association seems to be quite robust, since we adjusted for all possible confounders, and similar findings were obtained after multiple statistical comparative analyses. The NLR showed a moderate positive correlation with the worst EDSS. Multivariable regression analysis showed that lnNLR was associated with onset-to-sample time, lesion length, and the worst EDSS. Logistic regression analysis revealed that lnNLR was an independent factor predicting moderate to severe neurological disability (EDSS ≥ 4), as well as MRI lesion length and onset-to-sample time. However, further analysis with severe disability (EDSS ≥ 6) as the outcome suggested that MRI lesion length was the most important predictive factor among the variables. This was not surprising because we included only ITM without any brain lesions; therefore, EDSS was solely dependent on spinal lesions. In addition, NLR seemed to be significantly affected by the time delay of blood sampling from symptom onset, presumably because patients with moderate to severe disability or with an aggressive clinical course visited the emergency room earlier. We suppose that patients who couldn’t walk independently, which correlates with EDSS ≥ 6, would come earlier to the hospital. This would have affected the results of the correlation between NLR with worse EDSS. To avoid the above shortcomings, efforts were made to obtain the results of the blood test drawn in the acute phase at the primary center, but the white blood cell differential count was often unavailable in those cases. We believe that the above limitation should be considered when implementing NLR in future clinical research on ITM, which is probably not the case in disorders with clear disease onset (e.g., stroke, cardiovascular disease) or with high morbidity, which usually require regular care from a specialized center (e.g., MS, NMOSD).

The NLR is thought to represent a systemic inflammatory response, especially the involvement of the neutrophil-mediated innate immune system compared to the lymphocyte-mediated adaptive immune system, in the early active phase of the disease. The main pathophysiology of ITM is immune-mediated inflammation [22] and neutrophils play a pivotal role in acute inflammatory condition, like secreting pro-inflammatory or immunomodulatory cytokines. So, we predict that neutrophils are significantly involved in pathogenesis of ITM in acute phase, which would be related with NLR. Although previous studies have shown that NLR correlates with disease outcome in many immune-mediated CNS disorders, such as MS [8], NMOSD [10], and MOGAD [15], others have reported contradictory results [9, 11]. Hemond et al. [8] reported NLR could be related with neurological disability in MS, but applying NLR to future disability worsening must be careful due to its study design. On the other side, Gelibter et al. [9] showed no significant correlation of NLR with disease severity in MS and also asserts that different definition of disease activity and additional psychological symptoms related to this disorder may have resulted in contradictory results in previous studies. In NMOSD, Lin et al. [10] showed positive correlation of NLR in disease activity and additional neurological disability due to its role of neutrophil as a main inflammatory cell in NMOSD. However, Carnero Contentti et al. [11] revealed contrasting results. This study argues that several immunomodulatory agents could cause myelosuppression and NLR could be elevated in some chronic phases. It seems likely that the study results on MS or NMOSD would not be applicable to patients with ITM, whose characteristics were expected to be quite different. Most of our patients were treatment-naïve, monophasic ITM, which means that most of the patients had not been in a chronic inflammatory state prior to disease onset, in contrast to other patients with MS or other recurrent immune-mediated CNS demyelinating disorders. Another consideration should be that NLR is known to be affected by many factors, such as demographic factors (age, sex and ethnicity) [23], onset-to-sample time [13], psychological comorbidities [8], disease duration, disease subtype, and treatment [8, 24], which make it prone to change depending on the momentary condition of the patient. Lastly, caution should be exercised when generalizing our results, as NLR shows large differences across ethnic groups. A previous study investigating the reference value of NLR in a normal Asian population with more than 12,000 samples revealed an average NLR of 1.65 [23], in contrast to an average NLR of 2.24 in a non-Hispanic white population [25].

Our study had some limitations. This was a retrospective study from a tertiary university-affiliated hospital, which was subject to selection bias, including a referral bias. In addition, some data that might have affected NLR were not collected, such as smoking status and psychological comorbidities. Lastly, due to the lack of diagnostic biomarkers for ITM and the unavailability of serological assays for MOG antibody or CSF glial fibrillary acidic protein antibody in the past, the patients included in our study might have been heterogeneous. However, by thoroughly reviewing the medical records of all patients, we assured that none of the included patients complained of clinical symptoms suggestive of other CNS demyelinating disorders, such as MOGAD.

## Conclusion

We demonstrated that NLR may be used as an independent predictive marker for the worst neurological outcomes in patients with ITM. The findings in this study also revealed some flaws in NLR for practical use, such as a large dependency on the onset-to-sample time. To the best of our knowledge, this is the first study to investigate NLR in patients with ITM. We believe that this study provides directions for future research.

## Data Availability

The datasets used and/or analysed during the current study available from the corresponding author on reasonable request.
